# Pertussis and vaccination coverage in children under one year of age and in pregnant women: a challenge that persists

**DOI:** 10.1590/1984-0462/2026/44/2026054

**Published:** 2026-11-02

**Authors:** Paula Alves Alcalá, Letícia Bezerra Faria, Vitória Picolotti Elias, Maria Rita Donalisio

**Affiliations:** a Universidade Estadual de Campinas, Campinas, SP, Brazil.

**Keywords:** Whooping cough, Vaccination coverage, Pregnant people, Pertussis vaccine, Tetanus- diphtheria-acellular pertussis vaccines, Immunization programs, Coqueluche, Cobertura vacinal, Gestantes, Vacina contra coqueluche, Vacinas contra difteria, tétano e coqueluche acelular, Programas de imunização

## Abstract

**Objective:**

To analyze temporal trends in vaccination coverage for the third dose of the pentavalent/hexavalent vaccines among children under one year of age, Tetanus-diphtheria-acellular pertussis (Tdap) vaccination coverage among pregnant women, and pertussis incidence in children under one year of age in Campinas, state of São Paulo, Brazil, in the 2013-2025 period.

**Methods:**

This is an ecological, time-series study assessing vaccination coverage and pertussis incidence using data from the Brazilian National Information System. Segmented linear regression models were applied to estimate the Annual Percentage Change (APC) and respective 95% confidence interval (CI).

**Results:**

Pentavalent vaccination coverage exceeded 95% between 2013 and 2016, followed by a decreasing trend until 2019 (APC -4.03%; 95%CI -16.8; 9.2). From 2019 to 2025, results suggest an increasing trend (APC 3.08%; 95%CI -9.8; 18.3). As Tdap coverage increased from 15.82 to 80.4%, pertussis incidence in children under one year of age decreased from 37.8 to 8.6 per 10 thousand live births between 2013 and 2025, with an APC of -41.3% (95%CI -80.0; -21.4) from 2013 to 2022. In 2024, resurgence was observed across all age groups (135 cases), with an incidence of 27.1 per 10 thousand live births; 23% of cases in children under one year of age, with 96.8% confirmed by laboratory criteria.

**Conclusions:**

Recent recovery in vaccination coverage was observed in Campinas, and a substantial decline in pertussis incidence followed by the introduction of maternal Tdap vaccination. The resurgence of cases in 2024 underscores the ongoing vulnerability of the pediatric population and highlights the need to strengthen immunization strategies and epidemiological surveillance to prevent further increases in disease incidence.

## RESUMO

**Objetivo:**

Analisar tendências temporais da cobertura vacinal da terceira dose da vacina pentavalente/hexavalente em menores de um ano, da cobertura da vacina contra difteria, tétano e coqueluche acelular (dTpa) em gestantes e da incidência de coqueluche em menores de um ano em Campinas, São Paulo, no período de 2013-2025.

**Métodos:**

Estudo ecológico de séries temporais da cobertura vacinal e da incidência de coqueluche, com dados dos Sistemas de Informação em Saúde do Brasil. Modelos de regressão linear segmentada foram utilizados para estimar a Variação Percentual Anual (VPA) e os respectivos intervalos de confiança (IC) de 95%.

**Resultados:**

A cobertura da vacina pentavalente/hexavalente foi superior a 95% entre 2013 e 2016; os resultados sugerem tendência de queda até 2019 (VPA -4,03%; IC95% -16,8; 9,2). Entre 2019 e 2025, presume-se recuperação das coberturas (VPA 3,08%; IC95% -9,8; 18,3). Enquanto a cobertura da dTpa aumentou de 15,82 para 80,4%, a incidência de coqueluche em menores de um ano reduziu de 37,8 para 8,6 por 10 mil nascidos vivos entre 2013 e 2025, com uma VPA de -41,3% (IC95% -80,0; -21,1). Em 2024, observou-se recrudescimento da doença em todas as faixas etárias (135 casos), com incidência de 27,1 por 10 mil nascidos vivos; 23% dos casos em crianças menores de um ano e 96,8% nesta faixa foram confirmados por critério laboratorial.

**Conclusões:**

Observou-se recuperação da cobertura vacinal em Campinas e redução da incidência de coqueluche após a introdução da dTpa em gestantes. O aumento de casos em 2024 reforça a necessidade de fortalecer as estratégias de imunização e vigilância epidemiológica.

## INTRODUCTION

Pertussis (whooping cough) is a highly contagious respiratory infection caused by the gram-negative bacterium *Bordetella pertussis*. The clinical spectrum ranges from asymptomatic or mild cases to severe respiratory disease, with high hospitalization rates and significant impact on morbidity and mortality, particularly among infants under one year of age. Adults and adolescents often present with milder, underdiagnosed forms of the disease and may act as sources of infection for infants.^1^ ^,^ ^2^

Protective measures against pertussis include pediatric immunization with the pentavalent vaccine (diphtheria and tetanus toxoids, components of the *Bordetella pertussis*, *Haemophilus influenzae* type b antigens, and hepatitis B antigen) or hexavalent (including polio) and vaccination of pregnant women with the acellular pertussis-containing vaccine (Tdap). These vaccines are provided by the Brazilian National Immunization Program (*Programa Nacional de Imunizações* - PNI), coordinated by the Ministry of Health in cooperation with state and municipal health departments, and recognized as a global reference in immunization. The PNI plays a key role in maintaining high national vaccination coverage, reducing the incidence of vaccine-preventable diseases and protecting the population.^3^

Considering the epidemiological scenario of increasing pertussis cases since 2011, the Tdap vaccine for pregnant women was incorporated into the PNI vaccination schedule in 2014. Its main objective is to prevent transmission of the bacterium to the newborn and enable transplacental transfer of antibodies against *B. pertussis*, thereby protecting the infant until completion of the pentavalent/hexavalent vaccination during the first year of life, at two, four, and six months of age.^4^ ^,^ ^5^ ^,^ ^6^

 A decline In vaccination coverage for several vaccines recommended for children in their early years, including those protecting against pertussis, has been recorded in Brazil since 2016.^4^ Reduced immunization coverage increases the susceptible population, elevating the risk of resurgence of vaccine-preventable diseases, along with associated morbidity and mortality, and imposing additional strain on healthcare systems.^4^ ^,^ ^7^ ^,^ ^8^

In the final months of 2023 and early 2024, a notable global increase in cases of pertussis emerged.^9^ ^,^ ^10^ ^,^ ^11^ ^,^ ^12^ ^,^ ^13^ ^,^ ^14^ In Brazil, the state of Rio de Janeiro reported a 300% increase in cases by July 2024, compared with the total cases recorded in 2023. Similarly, the city of São Paulo experienced an alarming increase of 1,178% in confirmed cases by June 2024 in relation to the previous year.^11^ In Campinas, 135 confirmed cases of pertussis were registered in 2024, representing a significant increase compared to previous years.^15^

Considering the international and national increase in pertussis cases, monitoring vaccination coverage and disease incidence over time contributes to a better understanding of the impact of this epidemiological scenario. This approach enables the development and implementation of immunization strategies at the municipal level, aiming to protect the population against the pressure of introduction of vaccine-preventable diseases and strengthening epidemiological surveillance.

In the present study, we aimed to analyze temporal trends in vaccination coverage (VC) of the third dose of the pentavalent/hexavalent vaccine in infants, vaccination coverage of the Tdap vaccine in pregnant women, and the incidence of confirmed cases of pertussis in infants under one year of age in Campinas, state of São Paulo, Brazil, from 2013 to 2025.

## METHOD

This is an ecological study analyzing temporal trends from 2013 to 2025 of the incidence of pertussis, VC of the third dose of the pentavalent and hexavalent vaccine in infants under one year of age, and VC of the Tdap vaccine in pregnant women, after the 20th week of pregnancy in Campinas, São Paulo. The study follows the Reporting of studies Conducted using Observational Routinely-collected Data (RECORD) statement.

The study was conducted in the municipality of Campinas (approximately 1.2 million inhabitants according to the Brazilian Institute of Geography and Statistics [IBGE], 2022), located in the state of São Paulo and serving as a medical and health reference for the Metropolitan Region of Campinas.

Vaccination coverage of the pentavalent/hexavalent vaccine was calculated by dividing the number of third doses of the pentavalent vaccine (TDP/HepB/Hib) and the acellular hexavalent vaccine (TDaP/HepB/VIP/Hib/Polio inactivated) by the number of live births in the corresponding year, obtained from the Live Birth Information System (*Sistema de Informação Sobre Nascidos Vivos* - SINASC). The VC of the Tdap vaccine in pregnant women was calculated by dividing the total number of Tdap doses administered during that pregnancy by the number of live births in the corresponding year, obtained from SINASC. The target coverage for both vaccines is 95%.^6^

VC data were obtained from the public-access platform of the Information System for the National Immunization Program (*Sistema de Informações do Programa Nacional de Imunizações* - SI-PNI) from the Department of Informatics of the Brazilian Unified Health System (DATASUS).^16^

The incidence of confirmed pertussis cases was calculated by dividing the number of confirmed cases in children under one year of age by the number of live births in the corresponding year, multiplied by 10 thousand. Data were obtained from the Notifiable Diseases Information System (*Sistema de Informação de Agravos de Notificação* - SINAN) from confirmed cases of whooping cough in municipal residents. Confirmed cases were included in the study according to the Ministry of Health criteria: laboratory, clinical-epidemiological, or clinical:^17^ ^,^ ^18^

1. Laboratory criteria: cases that meet the definition of a suspected pertussis case and have isolation of *Bordetella pertussis* by culture or detection by Polymerase Chain Reaction (PCR);

2. Clinical-epidemiological criteria: cases that meet the definition of a suspected pertussis case (between those under and over six months of age) and had contact with a confirmed case according to laboratory criteria, between the onset of the catarrhal phase and up to three weeks after the onset of the paroxysmal phase;^17^

3. Clinical criteria: for infants under six months of age, cases that meet the definition of a suspect case in this age group and do not meet laboratory and/or clinical-epidemiological criteria; for individuals aged six months or older, cases that meet the definition of a suspect case in this age group and do not meet laboratory and/or clinical-epidemiological criteria.

Cases were classified annually according to laboratory diagnostic criteria. For the analysis of temporal trends in the VC of pentavalent/hexavalent vaccine in Campinas between 2013 and 2025, a segmented linear regression model was adjusted. After assessing normality, homoscedasticity, and absence of correlation in the data, Annual Percent Change (APC) and Average Annual Percent Change (AAPC) with 95% confidence intervals (CI) were estimated. The selected model was the one with the lowest weighted Bayesian Information Criterion (BIC). The analysis was performed using the Joinpoint Regression Program, version 4.5.0.1 and R v. 4.3.1.

The database analyzed in the article is anonymized and is available in an open repository of the Ministry of Health - DATASUS TABNET: Notifiable Diseases Information System - SINAN, Live Birth Information System - SINASC, and Information System for the National Immunization Program - SI-PNI. Ethical approval was not required as the database accessed contains open-access, de-identified, aggregated data (according to resolution No. 674/2022 of Ministry of Health/National Health Council - CNS).

## RESULTS

VC of the third dose of the pentavalent/hexavalent vaccine in Campinas was above the 95% target from 2013 to 2016, as well as from 2023 to 2025. However, coverage from 2017 to 2022 remained below this threshold, reaching a low of 72.7% in 2019 (Table 1). We observed a decreasing trend in VC from 2013 to 2019, with an APC of -4.0% (95%CI -16.8; 9.2) per year during the period. From 2019 to 2025, coverage increased, with an APC of 3.08% (95%CI -9.8; 18.3), reaching 98.1% in 2024, the highest level since 2016 (Figure 1; Table 1). The APC values obtained in the model were not statistically significant at the 0.05 level, although this model showed the lowest weighted BIC. Between 2013 and 2023, a decrease in the incidence of pertussis among children under one year of age was observed alongside an increase in Tdap vaccination coverage among pregnant women. From 2014 to 2015, the incidence of pertussis declined from 30.6 to 6.2 cases per 10 thousand live births (Figure 2). Over the period from 2013 to 2022, incidence consistently declined, with an APC of -41.3% per year, followed by an increasing trend from 2022 onward (Table 2). After a consistent drop in the pertussis incidence following the introduction of Tdap in 2014, incidence increased again in 2024, reaching 22.7 cases per 10 thousand live births (Table 1, Figure 2).

**Table 1.** Frequency of pertussis cases in infants under one year of age, percentage relative to total cases, and vaccination coverage of pentavalent/hexavalent and acellular pertussis-containing vaccine for pregnant women, Campinas, Brazil, 2013-2024.

Year 1st symptom	Frequency <1 year	Total cases	% cases <1 year/other	VC Pentavalent Hexavalent	VC Tdap pregnant women
2013	58	101	57.4	96.4	48.10
2014	64	155	41.3	99	2.44
2015	15	26	61.5	104.5	15.82
2016	16	39	41.0	105.2	61.62
2017	19	52	36.5	82.4	55.65
2018	20	50	40.0	90.9	36.64
2019	5	18	27.8	72.7	59.80
2020	1	3	33.3	92.4	62.22
2021	2	2	100.0	82.5	60.35
2022	0	1	0	87.5	60.34
2023	2	2	100.0	95.7	72.6
2024	31	135	23.0	98.1	83.9
2025	10	31	32.3	96.8	80.4

Note: Data from DATASUS.

VC: Vaccination coverage; Tdap: acellular pertussis-containing vaccine.

Source: DATASUS (https://datasus.saude.gov.br/)

**Table 2.** Estimators of the Annual Percent Change in the temporal trend of incidence of pertussis in children under one year of age residing in Campinas per 10 thousand live births, 2013 to 2025.

Annual Percent Change						
Segment	Lower endpoint	Upper endpoint	APC	Lower CI	Upper CI	p-value
1	2013	2022	-41.3*	-80.0	-21.4	<0.001
2	2022	2025	262.2	-6.6	1742	0.068

Notes: *Annual Percent Change is significantly different from zero at the alpha=0.05 level.

APC: Annual Percent Change; CI: confidence interval.

Source: DATASUS (https://datasus.saude.gov.br/)

**Figure 1.**
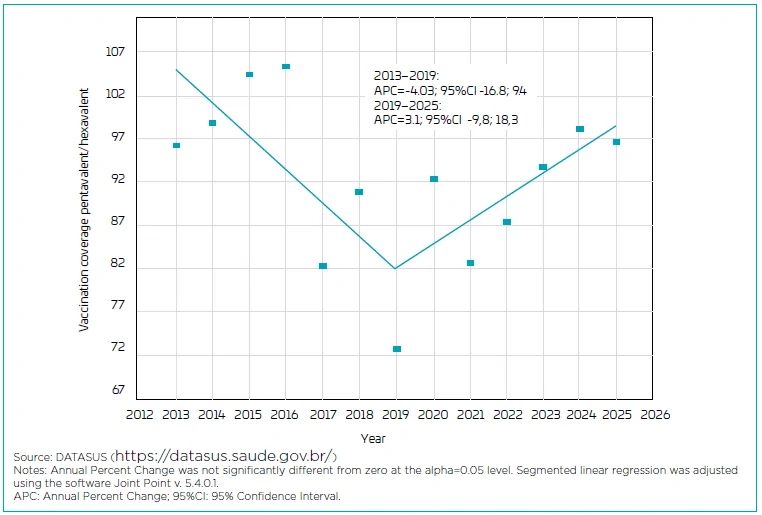
Temporal trend of vaccination coverage with the third dose of the pentavalent/hexavalent vaccine in children under one year of age, Campinas, Brazil, 2013 to 2025.

**Figure 2.**
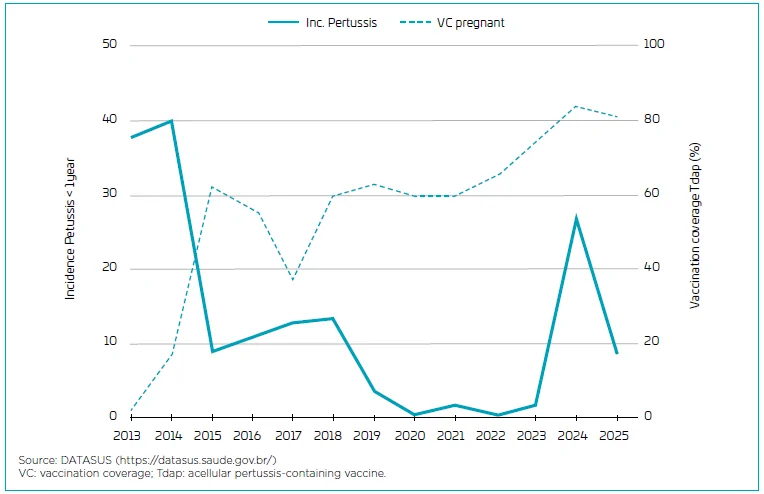
Vaccination coverage of the acellular pertussis-containing vaccine in pregnant women and incidence of pertussis in children under one year of age (per 10 thousand live births), Campinas, Brazil, 2013 to 2025.

Confirmed cases of pertussis among children under one year of age account for 39.5% of all cases among residents of Campinas between 2013 and 2025, although this proportion varied across the years of study (Table 1). Other age groups were also affected, particularly in the years of increased global incidence (2014 and 2024) (Table 1). The age-specific case registrations are summarized in supplementary material under request.

In 2024, 135 confirmed cases of pertussis were reported, of which 23.0% in individuals under one year of age (Table 1); 87 cases (80.6%) were confirmed by laboratory criteria, while 21 (19.4%) were confirmed based on clinical-epidemiological criteria. Considering only children under one year of age, 30 cases (96.8%) were confirmed by laboratory criteria, and one case (3.2%) was confirmed based on clinical-epidemiological criteria (Table 3).

**Table 3.** Criterion for confirming pertussis cases in infants under six months and between six and 12 months of age, Campinas, Brazil, 2013-2025.

Year 1st symptom	Cases <6 months	Laboratory Diag. Crit. ≤6 months %	Cases 6-12 months	*Laboratory Diag. Crit. 6-12 months (%)	Laboratory Criterion Total (%)	^†^ **Clinical/Epidem. Criterion Total (%)**
2013	46	42	4	8.7	46 (79.3)	12 (20.7)
2014	42	42	0	0.0	42 (65.6)	22(34.4)
2015	9	6	3	33.3	9 (56.3)	6 (37.5)
2016	12	12	0	0.0	12 (75.0)	4 (25.0)
2017	13	13	0	0.0	13 (68.4)	5 (26.3)
2018	17	15	2	11.8	17 (85.0)	3 (15.0)
2019	3	2	1	33.3	3 (60.0)	2 (40.0)
2020	1	1	0	0.0	1 (100.0)	0
2021	1	0	0	0	0	2 (100.0)
2022	30	0	8	0	0	0
2023	7	1	3	0.0	1 (50.0)	1 (50.0)
2024	46	22	4	26.7	30 (96.8)	1 (3.2)
2025	42	4	0	42.9	7 (30.0)	3 (70.0)

*Laboratory diagnostic criterion; ^†^Clinical or clinical/epidemiological criterion.

Source: DATASUS (https://datasus.saude.gov.br/)

## DISCUSSION

Pertussis VC in Campinas consistently declined as of 2017, reaching its lowest level in 2019, before resuming the recommended 95% target in 2023 and 2024. The progressive increase in Tdap coverage among pregnant women, following its introduction in 2014, although still below ideal levels, coincided with a reduction in pertussis incidence among children under one year of age.

A decline in VC has been observed nationally since 2016, particularly for the pentavalent vaccine, from 2011 to 2020.^4^ ^,^ ^19^ This trend was exacerbated during the COVID-19 pandemic. Between 2019 and 2020, Brazil experienced an 11.1% reduction in average VC among children under one year of age, reaching the lowest national average (75.1%) recorded between 2013 and 2020.^20^ According to the World Health Organization (WHO), by August 2020, 90% of 105 reporting countries had experienced disruptions in essential health services, with immunization among the most affected.^7^ Multiple factors contributed to this decline, including reduced access to health services due to social distancing measures, parental concerns about COVID-19 exposure, misinformation, health system resource constraints, supply chain disruptions, and vaccine hesitancy driven by political and social factors.^4^ ^,^ ^8^ ^,^ ^19^ ^,^ ^20^ ^,^ ^21^

In Brazil, pentavalent vaccine coverage was particularly affected. In Campinas, coverage reached a low of 72.7% in 2019 due to a supply crisis related to imported vaccine batches that did not meet regulatory standards of the Brazilian Health Regulatory Agency (*Agência Nacional de Vigilância Sanitária* - ANVISA).^4^ ^,^ ^20^ ^,^ ^22^ Nationally, coverage dropped to 70.8% in the same year, representing the largest decline among pediatric vaccines between 2015 and 2019.^4^ Although a national multi-vaccination campaign launched in October 2020 partially mitigated the decline, it did not fully compensate for previous losses.^23^ From 2021 onward, pentavalent coverage progressively improved in Campinas, reaching 98.1% in 2024, the first year since 2017 to surpass the 95% target.^16^ ^,^ ^17^

The introduction of maternal Tdap vaccination was associated with a marked reduction in pertussis incidence among infants under one year of age. In the first year following its introduction, despite limited coverage of only 61.6%, pertussis incidence in this age group decreased fivefold, from 30 to 6.2 cases per 10 thousand live births. Maternal immunization is highly effective in preventing severe disease and deaths in young infants through transplacental antibody transfer.^4^ ^,^ ^5^ ^,^ ^9^ Local coverage substantially increased, from 15% in 2014 to 80.4% in 2025, exceeding the national average in that period.^5^ The municipality’s extensive primary healthcare network may have contributed to improved adherence.

The marked reduction in reported and confirmed pertussis cases during 2020-2021 likely reflects both decreased circulation of respiratory pathogens due to non-pharmaceutical interventions and reduced health care utilization, which may have limited case detection and laboratory confirmation.^24^ Following this decline, a resurgence of pertussis was observed globally in 2024.^9^ ^,^ ^10^ ^,^ ^11^ ^,^ ^12^ ^,^ ^13^ ^,^ ^14^ In England, 2,793 confirmed cases were reported in the first quarter of 2024 compared to 858 cases in all of 2023, totaling 14,894 cases in 2024.^9^ ^,^ ^25^ In the United States of America, cases increased by approximately 300% in the first half of 2024. In July 2024, the Pan American Health Organization/World Health Organization issued an epidemiological alert in response to rising pertussis activity across the Americas.^12^

The recent increase in cases is likely multifactorial. The resumption of social interaction after the pandemic facilitated bacterial transmission. Enhanced clinical awareness, strengthened surveillance, and expanded use of molecular diagnostics may also have increased case detection.^13^ At the same time, VC remains heterogeneous and, in many regions, insufficient, particularly for pentavalent and maternal Tdap vaccines at the national level. Considering the efficacy of the *Bordetella pertussis* vaccine, protected individuals may be in smaller proportion compared to other vaccines, a phenomenon that justifies the importance of high VC for effective disease control. The cyclical pattern of pertussis (typically every three to five years), waning immunity, especially with cellular vaccines, migration of unvaccinated populations, and possible pathogen-related factors, can further contribute to the increase in cases. The emergence of *Bordetella pertussis* strains with genotypes differing from vaccine strains, suggesting potential vaccine escape, may also play a role. In addition, atypical and milder clinical presentations in adolescents and adults may hinder timely diagnosis, facilitating the transmission to vulnerable infants.^1^ ^,^ ^5^ ^,^ ^9^ ^,^ ^11^ ^,^ ^13^ ^,^ ^14^

In Campinas, 22.4% of cases were observed in children under one year of age (135 confirmed cases in total).^15^ Although the proportional burden shifted toward older age groups, this pattern remains concerning because adolescents and adults often present with milder or atypical symptoms, increasing the risk of transmission to infants, who are more likely to develop the severe disease.^2^ Similar age distribution shifts have been reported in other countries, including England, where most cases in 2024 occurred among individuals older than 15 years of age.^25^ The laboratory confirmation of 96.8% of the cases in infants under one year of age suggests that surveillance and medical assistance are alert in the face of a clinical condition with a challenging differential diagnosis, particularly during seasons (spring and winter) when other viral respiratory illnesses are circulating.

This study has limitations. The use of secondary data from national information systems may involve underreporting, inconsistencies, or data registration errors. Moreover, the multifactorial determinants of vaccination coverage and disease incidence limit causal inference, particularly in infants under one year of age. Nevertheless, this analysis is based on population-level data from a municipality with well-structured epidemiological surveillance and immunization systems. Health information systems remain essential tools for guiding public health decision-making and identifying priority groups. Even though the APC estimates were not significant at the 0.05 alpha level, the trends align with the vaccination coverage patterns observed in the model. Continued monitoring could ensure greater consistency to the results obtained in this study. Although these results are from the city of Campinas, similar trends can be identified in other municipalities across the country.

The resurgence of pertussis since 2024 is concerning, especially given the vulnerability of young children and the severity of the disease in infants. Vaccination remains the most effective strategy for long-term population protection. Strengthening epidemiological and molecular surveillance, restoring and sustaining high vaccination coverage, particularly among children and pregnant women, and continuous monitoring of pertussis incidence are essential to guide public health policies aimed at preventing further increases in cases and protecting vulnerable populations.

Despite the consistent recovery in vaccination coverage among infants under one year of age and pregnant women, pertussis remains an important public health concern requiring continuous epidemiological surveillance.

## Data Availability

The database that originated the article is available in an open repository (Department of Informatics of the Brazilian Unified Health System - DATASUS TABNET: Notifiable Diseases Information System - SINAN, Live Birth Information System - SINASC, and Information System for the National Immunization Program - SI-PNI) or with the corresponding author.
